# Investigation of Antibiotic Resistance and Biofilm Formation in Clinical Isolates of *Klebsiella pneumoniae*

**DOI:** 10.1155/2021/5573388

**Published:** 2021-06-14

**Authors:** Kiana Karimi, Omid Zarei, Parinaz Sedighi, Mohammad Taheri, Amin Doosti-Irani, Leili Shokoohizadeh

**Affiliations:** ^1^Student Research Committee, Hamadan University of Medical Sciences, Hamadan, Iran; ^2^Universal Scientific Education and Research Network (USERN), Tehran, Iran; ^3^Department of Microbiology, Faculty of Medicine, Hamadan University of Medical Sciences, Hamadan, Iran; ^4^Department of Epidemiology, School of Public Health and Research Center for Health Sciences, Hamadan University of Medical Sciences, Hamadan, Iran

## Abstract

**Aim:**

*Klebsiella pneumoniae* (*K. pneumoniae*) is an encapsulated Gram-negative bacterium that can lead to 14–20% of nosocomial infections. The ability of biofilm formation in this bacterium decreases the host immune response and antibiotic efficacy. This may impose a huge impact on patients and healthcare settings. This study aimed to evaluate the antibiotic resistance pattern and biofilm formation in *K. pneumoniae* strains isolated from two major Hamadan hospitals, west of Iran.

**Methods:**

A total of 83 *K. pneumoniae* strains were isolated from clinical samples of patients in different wards of Hamadan hospitals from September 2018 to March 2019. Determination of antimicrobial susceptibility was performed using the disk diffusion method. Biofilm formation was evaluated by the crystal violet method. Data were analyzed by the SPSS software and chi-square test.

**Results:**

The results showed that clinical samples included 18 urinary tract samples (22%), 6 wound samples (7%), 6 blood samples (7%), 17 tracheal tube aspiration samples (20%), 32 throat cultures (38%), 2 sputum samples (2.5%), and 2 abscess drain cultures (2.5%). High-level resistance to cefotaxime was detected in 92%, and all of isolates were susceptible to colistin. Biofilm formation was seen in 62 (75%) isolates. Strong biofilm formation was observed in 17 (20%) strains. A significant correlation was seen between biofilm formation and antibiotic resistance (*P* value <0.05).

**Conclusion:**

Our findings emphasize the need for proper diagnosis, control, and treatment of infections caused by *K. pneumoniae* especially in respiratory tract infections due to the strong biofilm formation and high antibiotic resistance in these strains.

## 1. Introduction

*Klebsiella pneumoniae (K. pneumoniae*) is a Gram-negative, encapsulated, nonmotile, rod-shaped bacterium and an important member of Enterobacteriaceae which can lead to various infections including gastrointestinal, skin, nasopharyngeal, osteomyelitis, biliary and urinary tract infections, and bacteremia. *K. pneumoniae* virulence factors including polysaccharide capsule which protects against bactericidal serum factors, type I and III pili that adhere to the surfaces, adhesins, and siderophore, play a key role in its pathogenicity [1, 2]. *K. pneumoniae* strain is more common in immunocompromised individuals such as diabetics and the elderly and children and widely colonized in hospitalized patients [3]. Urinary tract infection (UTI) is known to be the most prevalent type of nosocomial infection, and *K. pneumonia* is the second cause of urinary tract infections among other Gram-negative bacteria [4]. The increasing emergence of multidrug-resistant (MDR) bacterial pathogens is known as a major public health challenge worldwide [5]. The overuse of antibiotics has resulted in difficulties in the treatment of *K. pneumoniae* and limitations in our available options for effective management of this bacterial infection [4–6]. In Gram-negative bacteria, beta-lactam resistance has been detected through chromosomal or plasmid genes, but in clinical specimens, resistance is usually dependent on plasmid R genes [7, 8]. Extended-spectrum *β*-lactamase (ESBLs), part of group A beta-lactamases, can lead to the hydrolysis of broad-spectrum cephalosporin and lead to resistance to penicillin and cephalosporins. However, they can be inhibited by beta-lactamase inhibitors such as clavulanic acid [9, 10]. Previous studies have shown that *K. pneumoniae* strains that are resistant to a broad spectrum of antibiotics are rapidly expanding, especially when the bacteria are capable of forming biofilm [2]. These bacteria can form a thick layer of extracellular biofilm which helps them attach to living and abiotic surfaces [11]. Treatment of infections caused by biofilm-forming *K. pneumoniae* strains is more difficult than other strains [12]. The antibiotic resistance and bacterial tendency to biofilm formation may play a key role in the emergence of MDR-*K. pneumoniae* strains [13]. Due to the antiphagocytic feature of biofilm, it is more challenging for the host immunity to eliminate this kind of bacterial pathogens [14]. The use of antibiotics in patients with bacterial infections can lead to bacterial elimination and accelerate the treatment process. Consequently, the increasing incidence of drug resistance causes complications in patients and higher medical costs [15]. This study was designed to investigate the biofilm formation and antibiotic resistance patterns in clinical isolates of *K. pneumoniae* isolated from Hamadan hospitals, west of Iran.

## 2. Methods

This study was performed on 83 *K. pneumoniae* strains isolated from clinical samples of patients with various infections in different wards of Hamadan hospitals from September 2018 to March 2019. The isolates were identified as *K. pneumoniae*, using conventional microbiological tests [16]. After recognition, the *K. pneumoniae* strains were stored in trypticase soy broth (TSB) containing 18% glycerol at −70°C.

Antimicrobial susceptibility to 15 different antibiotics including amikacin (30 *µ*g), ampicillin-sulbactam (10/10 *µ*g), trimethoprim-sulfamethoxazole (1.25 + 23.75 *µ*g), ceftriaxone (30 *µ*g), cefotaxime (30 *µ*g), ceftazidime (30 *µ*g), ciprofloxacin (5 *µ*g), levofloxacin (5 *µ*g), imipenem (10 *µ*g), meropenem (10 *µ*g), colistin (10 *µ*g), gentamicin (10 *µ*g), piperacillin-tazobactam (100/10 *µ*g), tobramycin (10 *µ*g), and nitrofurantoin (300 *µ*g) was detected by the disk diffusion method according CLSI criteria [17]. The antibiotic disks were supplied by (Rosco Co., Denmark). *Escherichia coli* ATCC 25922 was used as the quality control strain. MICs of colistin in *K. pneumoniae* isolates were determined by the broth microdilution method over a range of dilutions from 0.125 to 128 *μ*g/ml using colistin sulfate (Sigma–Aldrich). According to European Committee on Antimicrobial Susceptibility Testing (EUCAST) guidelines [18, 19], breakpoints were used for interpretation of colistin MIC results (susceptible, ≤2 *µ*g/ml; resistant, >2 *µ*g/ml).

The biofilm formation was performed by the microtiter plate method (MTP) as described previously. The ability of the biofilm formation in each test isolate was compared with the negative and positive controls by analyzing the absorbance of the crystal violet stain applied for each biofilm. The following values were allocated for definition of the biofilm formation: nonbiofilm producer: OD550 ≤ 1, weak biofilm producer: 1 < OD550 ≤ 2, medium biofilm producer: 2 < OD550 ≤ 3, and strong biofilm producer: OD595 > 3 [20, 21].

SPSS software (version 22) was used for statistical analysis. Chi-square analysis was used for comparisons between the capacity of biofilm production and antibiotic resistance.

## 3. Results

Out of 83 *K. pneumoniae* clinical isolates, 32 (38.5%) were collected from the throat culture, 18 (21.6%) from the urine, 17 (20.4%) from the trachea, 6 (7.25%) from the blood culture, 6 (7.25%) from the wound, 2 (2.5%) from the sputum, and 2 (2.5%) from the abscess drain samples. About 57 (68%) of *K. pneumoniae* strains were obtained from male and 26 (32%) from female patients. Among all clinical isolates, 9 (11%) were taken from outpatients and 74 (89%) from inpatients specimens.

**Figure 1:**
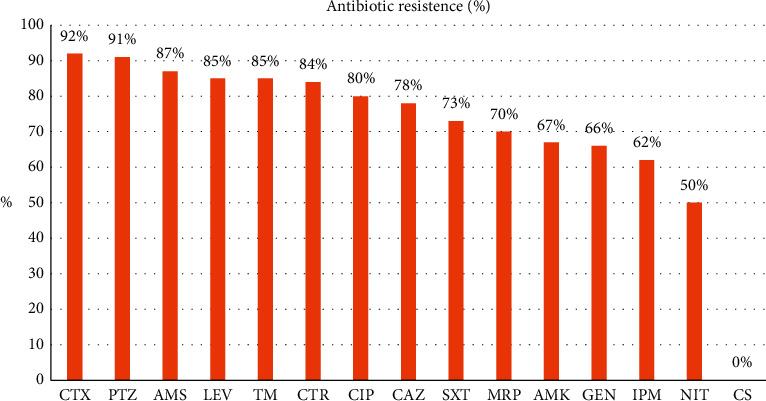
The prevalence (%) of resistance to antibiotics in *K. pneumoniae* isolates. CS, colistin; NIT, nitrofurantoin; IPM, imipenem; GEN, gentamicin; AMK, amikacin; MRP, meropenem; SXT, trimethoprim-sulfamethoxazole; CAZ, ceftazidime; CIP, ciprofloxacin; CTR, ceftriaxone; TM, tobramycin; LEV, levofloxacin; AMS, ampicillin-sulbactam; PTZ, piperacillin-tazobactam; CTX, cefotaxime.

According to antibiogram results of 83 clinical isolates of *K. pneumoniae*, the highest antibiotic resistance was related to cefotaxime (92%), and all isolates were susceptible to colistin. After cefotaxime, the most resistance was to piperacillin-tazobactam (91%) and ampicillin-sulbactam (87%). Resistance to levofloxacin and tobramycin was detected in 85% of isolates. The prevalence of resistance to ceftazidime, trimethoprim-sulfamethoxazole, amikacin, ciprofloxacin, meropenem, imipenem, gentamicin, and ceftriaxone are shown in Figure 1. Different antibiotic resistance patterns were detected in various clinical samples, for example, the highest resistance to amikacin, ceftazidime, and gentamicin was found in wound and blood samples and the highest antibiotic resistance to trimethoprim-sulfamethoxazole, tobramycin, cefotaxime, piperacillin-tazobactam, ampicillin-sulbactam, and meropenem was observed in urine samples.  Table 1 provides the prevalence of antibiotic resistance in different clinical specimens. A significant correlation was seen between the type of specimen and antibiotic resistance (*P* value <0.05). All *K. pneumoniae* isolates were susceptible to colistin, and the colistin MICs of 63 (75%), 11 (%13), and 9 (12%) of *K. pneumoniae* isolates were 0.125 *µ*g/ml, 0.5 *µ*g/ml, and 1 *µ*g/ml, respectively.

Our findings indicate that 62 (74.5%) *K. pneumoniae* isolates formed biofilm. The strains were classified into four categories as described above. Based on the biofilm analysis, 27 (32.5%) *K. pneumoniae* isolates formed biofilm weakly, 18 (21.6%) isolates created moderately, and 17 (20.4%) isolates were strong producers of biofilms. Among different clinical specimens, the lowest biofilm formation was seen in drainage abscess, and sputum culture and endotracheal aspiration samples had the highest biofilm formation. Additionally, blood culture samples formed biofilm weakly more than others and aspiration of the endotracheal tube showed the highest moderate biofilm formation. A significant correlation was seen between the type of clinical sample and the rate of biofilm formation (*P* value = 0.000), and the results are given in Table 2. Statistical analysis showed that there is a meaningful correlation between biofilm formation and resistance to antibiotics (*P* value <0.05). The results are given in Table 3.

## 4. Discussion

In this current research, the ability of biofilm formation in *K. pneumoniae* isolates obtained from the clinical specimens and the correlation between the strength of biofilm formation and patterns of antibiotic resistance, sites of infection, or type of clinical samples were assayed. Our findings indicated that high percentages (74.5%) of *K. pneumoniae* isolates were able to form biofilms, and 20.4% of them formed biofilm strongly. It has already been determined that the formation of biofilms by bacteria is linked to the development of infections associated with implants and catheters which can even threaten the lives of patients with cystic fibrosis and chronic wound infections [22]. *K. pneumoniae* is now considered a biofilm-forming bacterium that can lead to nosocomial opportunistic infections and also affects the efficacy of antibiotic treatments [23, 24].

There are different findings of the correlation between biofilm formation and site of infection. Yang and colleagues conducted an investigation on biofilm formation by *K. pneumoniae* strains isolated from blood samples, wounds, swabs, urine, and sputum samples. Their study indicated that 62.5% of the isolates generated biofilms, which is less than our results [24]. Seifi et al. in Iran reported that 93.6% of *K. pneumoniae* isolates had the ability of biofilm formation and 33% of them could produce biofilm strongly which is more than the results of our study. They also reported that strong biofilms were more common in wound and sputum specimens, while in our study, the samples collected from the tracheal tube were able to form stronger biofilms, and sputum specimens formed weaker biofilm compared to them [11]. Diversities in the results of different studies can be related to the geographical area, type, and number of samples or characteristics of bacterial isolates, including antibiotic resistance patterns.

Another result of this study was that the amount of *K. pneumoniae* isolates in men was higher than in women (68%). Some studies, such as one by Nirwati et al. revealed that in 64% of cases*, K. pneumoniae* was isolated from male patients [25], and also, almost the same results were obtained in studies conducted by Osagei et al. from Nigeria and Akter et al. from Bangladesh [26, 27]. A possible reason for the high risk of developing *K. pneumoniae* infections in men could be the higher consumption of cigarettes and alcohol in men compared to women [25].

In the present study, isolates related to the respiratory system were more than isolates extracted from urine culture and blood samples, consistent with the studies of Yang et al. from China and Nirwaty et al. from Indonesia [24, 25]. However, according to research by Seifi et al. from Iran, the isolates from urine culture (61.7%) were higher than the wound, blood, and sputum samples [11].

Our findings reveal that over 90% of isolates were resistant to cefotaxime and piperacillin-tazobactam, and also, more than 80% of strains were resistant to ampicillin-sulbactam, levofloxacin, tobramycin, ceftriaxone, and ciprofloxacin. Furthermore, more than 70% of isolates were resistant to ceftazidime and trimethoprim-sulfamethoxazole. Colistin was the only effective antibiotic. This is in contrast to the findings of Borges et al. who have identified meropenem and piperacillin-tazobactam as effective antibiotics against *K. pneumoniae* [23]. In our study, the resistance to ciprofloxacin is higher than the resistance reported by Madahiah et al. and Cepas et al. [28, 29]. According to the results of a research from Iran, the highest antibiotic resistance in *K. pneumoniae* isolates belongs to ampicillin (91%), the lowest resistance is related to imipenem (5.5%), and more than 50% of the strains were resistant to ceftazidime and cefalotin [30]. However, our results indicate that 62% of isolates were resistant to imipenem, and almost 73% of specimens were resistant to ceftazidime.

Another finding of the study was that the strength of biofilm formation in antibiotic-resistant strains was higher than the sensitive strains, and a remarkable correlation was observed between antibiotic resistance and biofilm formation. However, among sensitive isolates, biofilm formation has been observed, or in resistant strains, nonbiofilm producers and weak and moderate biofilm former have been observed. Different results have been reported in this regard. Zheng et al. have demonstrated no correlation between biofilm formation and the production of broad-spectrum beta-lactamase (ESBL) enzymes in the *K. pneumoniae* strains [14]. According to Zheng et al. and Yang's and Zhang study, about 80% percent of the *K. pneumoniae* strains, which were extracted from urine and sputum samples and tested positive for biofilm, were also able to produce ESBL [14, 24]. It has also been claimed that there may be a correlation between the strength of biofilm formation in bacterial strains and different geographical areas. One of the most important limitations of this study was the almost insufficient number of *K. pneumoniae* isolates from patients, due to limited supply of materials for laboratory tests and limitations in financial support. More sampling and molecular epidemiological studies are recommended to obtain stronger results.

In conclusion, results of this study confirm the role of biofilm formation in resistance to clinical isolates of *K. pneumoniae* isolated from Hamadan hospitals, west of Iran. The strength of biofilm formation was different in clinical samples. So, characterization of nosocomial pathogens is very useful to control and treat infections caused by these pathogens.

## Figures and Tables

**Table 1:** Prevalence of antibiotic resistance in *K. pneumoniae* isolated from different clinical samples.

Antibiotic	CS (%)	NIT (%)	IPM (%)	GEN (%)	AMK (%)	MRP (%)	SXT (%)	CAZ (%)	CIP (%)	CTR (%)	TM (%)	LEV (%)	AMS (%)	PTZ (%)	CTX (%)
Sample type															
Urine	0	50	40	45	42	100	100	75	62	87	100	—	100	100	100
Wound	0	—	83	100	83	83	66	83	100	—	83	100	80	80	66
Blood	0	—	50	100	100	50	60	100	80	—	100	75	100	100	—
Throat	0	—	65	100	70	64	62.1	70	83	—	82	81	93	93	100
Sputum	0	—	100	—	0	100	—	—	0	0	—	—	0	—	—
Trachea	0	—	100	75	80	100	100	100	90	100	100	100	92	75	—
Abscess	0	—	0	—	—	0	100	0	100	—	0	100	0	100	—

CS, colistin; NIT, nitrofurantoin; IPM, imipenem; GEN, gentamicin; AMK, amikacin; MRP, meropenem; SXT, trimethoprim-sulfamethoxazole; CAZ, ceftazidime; CIP, ciprofloxacin; CTR, ceftriaxone; TM, tobramycin; LEV, levofloxacin; AMS, ampicillin-sulbactam; PTZ, piperacillin-tazobactam; CTX, cefotaxime.

**Table 2:** Biofilm formation in *K. pneumoniae* isolated from different clinical samples.

Sample type	Biofilm formation strength			
	No biofilm (%)	Weak (%)	Moderate (%)	Strong (%)
Urine	29.0	35.0	11.0	23.0
Wound	33.0	50.0	00.0	16.0
Blood	20.0	60.0	00.0	20.0
Throat	25.0	35.0	25.0	13.0
Sputum	100	00.0	00.0	00.0
Trachea	12.0	18.0	31.0	37.0
Abscess	100	00.0	00.0	00.0

**Table 3:** Comparison of biofilm formation in antibiotic-resistant *K. pneumoniae* strains.

Antibiotics	Strength of biofilm formation						
		Number	Negative	Poor	Moderate	Strong	*P* value
Ampicillin-sulbactam	Resistant	54	10	17	15	12	0.038
	Susceptible	8	5	2	0	1	

Cefotaxime	Resistant	12	4	5	0	3	<0.001
	Susceptible	1	0	1	0	0	

Ceftazidime	Resistant	45	10	15	11	9	<0.001
	Susceptible	11	5	4	1	1	

Ceftriaxone	Resistant	11	4	2	2	3	<0.001
	Susceptible	1	1	0	0	0	

Piperacillin-tazobactam	Resistant	45	11	20	7	7	<0.001
	Susceptible	3	1	2	0	0	

Tobramycin	Resistant	41	8	16	8	9	0.035
	Susceptible	7	5	1	1	0	

Ciprofloxacin	Resistant	62	14	19	17	12	<0.001
	Susceptible	14	5	7	0	2	

Levofloxacin	Resistant	35	9	11	10	5	<0.001
	Susceptible	6	3	3	0	0	

Trimethoprim-sulfamethoxazole	Resistant	8	4	5	0	3	<0.001
	Susceptible	15	5	4	5	1	

Imipenem	Resistant	39	9	15	10	5	<0.001
	Susceptible	22	10	7	1	4	

Meropenem	Resistant	36	8	14	9	5	<0.001
	Susceptible	15	6	5	1	3	

Gentamicin	Resistant	14	3	8	2	1	<0.001
	Susceptible	7	3	2	0	2	

Amikacin	Resistant	44	5	16	10	13	0.024
	Susceptible	19	10	5	1	3	

Nitrofurantoin	Resistant	4	3	1	0	0	<0.001
	Susceptible	3	2	1	0	0	

## Data Availability

The data used to support the findings of this study are included within the article.

## Abbreviations

*K. pneumoniae*: *Klebsiella pneumoniae*
MDR: Multidrug-resistant
ESBL: Extended-spectrum *β*-lactamase
TSB: Trypticase soy broth
MTP: Microtiter plate method
CTX: Cefotaxime
PTZ: Piperacillin-tazobactam
AMS: Ampicillin-sulbactam
LEV: Levofloxacin
TM: Tobramycin
CTR: Ceftriaxone
CIP: Ciprofloxacin
CAZ: Ceftazidime
SXT: Trimethoprim-sulfamethoxazole
MRP: Meropenem
AMK: Amikacin
GEN: Gentamicin
IMP: Imipenem
NIT: Nitrofurantoin
CS: Colistin.
